# Supplementary material for: Exploratory study of risk factors related to SARS-CoV-2 prevalence in nursing homes in Flanders (Belgium) during the first wave of the COVID-19 pandemic
Source: PLoS One. 2023 Oct 5;18(10):e0292596. doi: 10.1371/journal.pone.0292596 (PMC10553833; doi:10.1371/journal.pone.0292596)
Supplement: S1 File — (DOCX) [file pone.0292596.s001.docx]

Dear director,

Dear manager,

This questionnaire is offered to you before the sampling within your institution in order to give you time to collect the data and speak to the appropriate persons for this purpose.

We are going to ask you a number of questions about the specific characteristics of the care facility (infrastructure, number of beds, etc.), the residents and staff, the severity with which the facility was affected during the lockdown and the measures you may have taken.

At several points in the questionnaire you are asked to give a date when certain measures were implemented: we realize that it is virtually impossible to give an exact date, so we ask you to fill in this date 'approximately'.

It is important for the research that you try to fill in all these questions as honestly as possible. Only in this way can we gain insight into what factors were important for the infection and spread of the Covid-19 virus within the nursing homes.

We would like to emphasize that the data are pseudonymized. The researchers have no insight into the relationship with the identity of the rest homes. A trusted third party will vouch for code per participating rest homes and thus have the link with the identity of the rest homes and manage this list as stipulated in the GDPR law.

Thank you in advance for your time and cooperation!

The researchers from Liantis and Ghent University

Liantis: Tom Geens, Heidi Janssens & Marc Borguet

Ghent University: Stefan Heytens & Piet Cools

There are 67 questions in this survey.

1. **Prior info**
2. You have probably already been informed that your nursing home will be contacted by a Liantis nurse for the practical organization and logistics of the sampling.

In order for this to go smoothly, we ask you to provide below the contact details of some of your collaborators.

The results of this question, after conducting the fieldwork and collecting all the data, will be removed from the database.

*Answer:*

1. **Your facility**

The questions below probe some general characteristics of your facility

1. What is the type of your facility? *

*Choose one of the following answers*

- Private facility
- Public facility
- NPO

1. How many beds did your facility have on February 1, 2020?

By this we mean all beds except those in service flats: so beds in both retirement and nursing homes & residential care facilities and short-stay beds.

Only numbers may be entered in this field.

*Please enter your answer here:*

1. How many residents (do NOT include service apartment residents) did your facility have on February 1?

Only numbers may be entered in this field.

*Enter your answer here*:

1. How many employees (counted in heads) were actively part of your organization (not only employees, but also self-employed, volunteers, interns,...) on Feb. 1?

Only numbers may be entered in this field.

*Enter your answer here*

1. What is the degree of urbanization of the area in which your facility is located? *

Choose one of the following answers

- high (city)
- middle (peripheral)
- low (rural)

1. What is the year of construction of your facility's (main) building?

Only a whole number may be entered in this field.

*Enter your answer here:*

1. Has the building already been renovated? *

*Choose one of the following options:*

- Yes
- No

1. In what year did this renovation occur?

*Answer this question only if the following conditions are met:*

*Answer was 'Yes' to question '8 [Renovation]' (Was the building already renovated?).*

Only numbers may be entered in this field.

*Please enter your answer here:*

1. What type of renovation was involved? *

*Answer this question only if the following conditions are met:*

*Answer was 'Yes' to question '8 [Renovation]' (Was the building already renovated?).*

*Multiple answers possible*

- Choose all the options that apply to you:
- Renovation in the context of improving energy efficiency
- Extensive renovation works (roof, windows,...)
- Less extensive renovation/embellishment
- Other:

1. Does the facility consist of several buildings?

Will separate buildings in which only service apartments or day care centers are located do NOT count.*

*Choose one of the following options:*

- Yes
- No

1. How many buildings does your facility consist of?

Do NOT want to include separate buildings that contain only service apartments or day care centers.

*Answer this question only if the following conditions are met:*

*Answer was "Yes" to question "11 [Nlocations]" (Does the facility consist of several buildings?*

*Do NOT want to count separate buildings in which only service flats or day care centers are located.)*

Only numbers may be entered in this field.

*Enter your answer here:*

1. How many living groups (cluster of residents, covered by 1 head nurse/coordinator) does your facility consist of?*

Only numbers may be entered in this field.

*Enter your answer here:*

1. Did your nursing home have a separate ward for residents with dementia on Feb. 1? *

*Choose one of the following options:*

- Yes
- No

1. Did your retirement home have an active ventilation system on Feb. 1? *

*Choose one of the following answers*

- no, there is only passive ventilation with the possibility of opening windows and doors
- no, there are grilles in the windows, but there is no air extraction
- no, but there is air cooling/heating possible apart from any active ventilation system
- yes, there is an extraction system in combination with grilles in the windows
- yes, there is balanced ventilation with (partial) recirculation of air
- yes, there is a balanced ventilation system that only uses fresh outside air (without recirculation)

1. Were rooms structurally naturally ventilated (by opening doors and windows) at the beginning (first weeks) of the Covid-19 epidemic? *

*Choose one of the following answers*

- yes, through opening windows
- yes, via opening of doors
- yes, via opening windows and doors
- No

1. Were common areas structurally naturally ventilated (via opening windows and doors) at the beginning (first weeks) of the epidemic? *

*Choose one of the following answers*

- yes, structurally and continuous ventilation was provided
- yes, at regular times (e.g. after increased use of the room)
- No

1. **To what extent was your facility affected?**

We will ask you a number of questions about residents as well as employees. We ask that you focus specifically on those residents and employees who were already residing or working in the facility on 1/2/2020.

1. How many of the residents, who resided in the facility on 1/2/2020 (including those who have since died), have been ill (had typical Covid symptoms^1^) since the beginning of the epidemic to the present?

Only numbers may be entered in this field.

*Enter your answer here:*

^1^*A possible case of COVID-19 is a person with (source: sciensano case definition)*

*at least one of the following chief symptoms that arose acutely, with no other apparent cause :*

*-cough; shortness of breath; thoracic pain; acute loss of smell and/or taste;*

*OR*

*-at least two of the following symptoms, with no other apparent cause,*

*fever ; muscle pain ; fatigue ; rhinitis ; sore throat ; headache ; anorexia ; watery diarrhea ; acute confusion ; sudden fall ;*

*OR*

*-Exacerbation of chronic respiratory symptoms (COPD, asthma, chronic cough...), without other obvious cause.*

1. Since the beginning of the corona epidemic, how many of the residents residing in the facility on 1/2/2020 have tested positive for a PCR nose and/or throat swab since the beginning of the corona epidemic to date (we are counting the number of residents here, not the number of tests!)?

Only numbers may be entered in this field.

*Enter your answer here:*

1. Since the start of the corona epidemic, how many residents, who resided in the facility on 1/2/2020, have died from Covid-19 (after proven positive tests) since the start of the corona epidemic until now?

Only numbers may be entered in this field.

*Enter your answer here:*

1. Since the beginning of the corona epidemic, how many residents, who resided in the facility on 1/2/2020, have died with a suspected Covid-19 (i.e., without a positive test) since the beginning of the corona epidemic until now?

Only numbers may be entered in this field.

*Enter your answer here:*

1. How many employees (including self-employed and volunteers), who were active in the facility on 1/2/2020, have had the typical Covid-19 symptoms^1^ (i.e., also suspected Covid-19), since the beginning of the Covid-19 epidemic to the present?

Only numbers may be entered in this field.

*Enter your answer here:*

^1^*A possible case of COVID-19 is a person with (source: sciensano case definition)*

*at least one of the following chief symptoms that arose acutely, with no other apparent cause :*

*-cough; shortness of breath; thoracic pain; acute loss of smell and/or taste;*

*OR*

*-at least two of the following symptoms, with no other apparent cause,*

*fever ; muscle pain ; fatigue ; rhinitis ; sore throat ; headache ; anorexia ; watery diarrhea ; acute confusion ; sudden fall ;*

*OR*

*-Exacerbation of chronic respiratory symptoms (COPD, asthma, chronic cough...), without other obvious cause.*

1. How many employees, active in the facility on 1/2/2020, have tested positive for a PCR nose and/or throat swab, since the beginning of the corona epidemic to the present?

Only numbers may be entered in this field.

*Enter your answer here:*

1. How do you estimate the number of sickness absences from your staff since the beginning of the corona epidemic to the present? *

*Choose one of the following answers*

- Higher than previous years
- Same as previous years
- Lower than previous years

1. **Demographic characteristics and severity of care**

Here we ask again to remember the situation as it was at the beginning of the Covid pandemic.

1. How many of the residents and staff, who resided or were active in the facility on 1/2/2020, are women ?

Only numbers may be entered in these fields.

Number of female residents:

Number of female employees (including self-employed and volunteers)

1. How many of the residents (not including residents of the service apartments and day care centers) who resided in the facility on 1/2/2020 are or were 85 years of age or older?

Only numbers may be entered in this field.

*Enter your answer here:*

1. On 1/2/2020, what was the distribution of resident dependency categories (please express in number of residents)?

|  | Number of residents (in february) |
| --- | --- |
| A |  |
| B |  |
| C |  |
| Cd |  |
| D |  |

1. **Prevention measures**

The following questions probe specifically about the measures that were taken. For some measures, we also ask you to estimate when (approximately) this measure was introduced.

1. From approximately when did you start taking specific Covid-19 measures on top of pre-existing (everyday) precautions?*

*Choose one of the following answers*

- first half of February
- second half of February
- first half of March
- second half of March
- first half of April
- second half of April
- first half of May
- second half of May
- first half of June
- second half of June
- no additional measures taken

1. At the beginning of the Covid-19 epidemic, did you have the ability to isolate infected residents (contact drop isolation) from the rest of the residents? *

*Multiple answers possible*

- Choose all options that apply to you:
- yes, by contact drop isolation in the resident's own room
- yes, by cohort care at the ward level, per floor or part of the ward/floor
- yes, by cohort care in common areas
- yes, but for this we had to cooperate with other facilities
- no, we did not have that possibility

1. During the Covid-19 epidemic, did you effectively isolate infected residents from uninfected residents ? *

*Answer this question only if the following conditions are met:*

*Answer was to question '29 [IsolationBew]' (Did you, at the beginning of the Covid-19 epidemic, have the ability to isolate infected residents (contact drop isolation) from the rest of the residents?)*

*Choose one of the following answers*

- yes
- no
- not applicable because there were no infected residents

1. From when did you begin applying these isolation measures to residents (approximately)?

*Answer this question only if the following conditions are met:*

*Answer was 'yes' to question '30 [IsolationApplied]' (During the Covid-19 epidemic, did you effectively isolate infected residents from uninfected residents ?).*

*Enter a date:*

1. Since the start of the Covid-19 epidemic, was there a requirement for residents to wear mouth masks? *

*Choose one of the following options:*

- Yes
- No

1. Approximately when was this mouth mask requirement for residents introduced?

*Answer this question only if the following conditions are met:*

*Answer was "Yes" to question "32 [Mouth MaskBew]" (Since the beginning of the Covid-19 epidemic, was mouth mask requirement introduced for residents?).*

*Enter a date:*

1. What type of mouth masks were used on residents?

*Answer this question only if the following conditions are met:*

*Answer was "Yes" to question "32 [Mouth MaskBew]" (Since the beginning of the Covid-19 epidemic, was mouth mask mandatory for residents?).*

*Multiple answers possible*

*Please select all the options that apply to you:*

- surgical mask
- cloth mask

1. Since the beginning of the Covid-19 epidemic, were mouth mask requirements implemented for employees? *

*Choose one of the following options:*

- Yes
- No

1. From when (approximately) was this mouth mask requirement for employees implemented?

*Answer this question only if the following conditions are met:*

*Answer was "Yes" to question "35 [Mouth MaskMedew]" (Since the beginning of the Covid-19 epidemic, was mouth mask requirement introduced for employees?).*

*Enter a date:*

1. What type of mouth masks were used by employees?

*Multiple answers possible*

*Please choose all the options that apply to you:*

- surgical mask
- cloth mask
- P2 filter mask

1. Prior to the outbreak of the Covid-19 epidemic, was hand disinfectant available to staff at several sites (near each room, on care carts, nursing stations, etc.)?*

*Choose one of the following options:*

- Yes
- No

1. Was the availability of hand disinfectant for employees at various sites increased since the outbreak of the epidemic? *

*Choose one of the following options:*

- Yes
- No

1. From when (approximately) was the availability of hand alcohol gel to employees increased?

Answer this question only if the following conditions are met:

Answer was "Yes" to question "39 [Alcogelmedewopgevoerd]" (Was the availability of hand sanitizer for employees at various sites ramped up since the outbreak of the epidemic?).

*Enter a date:*

1. Was hand disinfectant available to residents during the Covid-19 epidemic? *

*Choose one of the following options:*

- Yes
- No

1. Before the Covid-19 epidemic broke out, was hand disinfectant available to visitors? *

*Choose one of the following options:*

- Yes
- No

1. From the beginning of the corona epidemic, were the rooms cleaned and disinfected daily with a product active against Sars-CoV-2 ? *

*Choose one of the following options:*

- Yes
- No

1. Was the measure implemented at a later date to clean and disinfect the rooms daily with a product active against Sars-CoV-2? *

*Answer this question only if the following conditions are met:*

*Answer was "No" to question "43 [Cleaning]" (From the beginning of the corona epidemic, were the rooms cleaned and disinfected daily with a product active against Sars-CoV-2 ?).*

*Choose one of the following options:*

- Yes
- No

1. From approximately when were rooms cleaned and disinfected daily with an agent active against Sars-CoV2?

*Answer this question only if the following conditions were met:*

*Answer was 'Yes' to question '44 [CleaningAct]' (At a later date, was the measure implemented to clean and disinfect rooms daily with a product active against Sars-CoV-2?)*

*Enter a date:*

1. Since the beginning of the Covid-19 epidemic, what was the first measure taken, vs. visitation?

*Choose one of the following answers*

- No more visitors allowed
- Visit of limited number or 1 person
- Visit in separate location with physical separation
- Visit limited in time

1. From when (approximately) was the first specific measure vs. the visit applied?

*Enter a date:*

1. From approximately when were events and activities with externals discontinued?

*Enter a date:*

1. Which was the first specific measure regarding meals with residents that was implemented? *

*Choose one of the following answers*

- Residents eat together but with social distancing
- Residents eat separately

1. From when (approximately) was this first meal-taking measure applied to residents?

*Enter a date:*

1. Approximately when was the first round of PCR testing conducted on residents (and employees) organized by the government?

*Enter a date:*

1. Did the facility have the necessary testing capacity when needed? *

*Choose one of the following answers*

- yes, but only on its own resources
- yes, with the government test rounds
- yes, with the government test rounds and on own resources
- No

1. Did the facility itself organize PCR testing with its own resources even before the testing came from the government? *

*Choose one of the following answers*

- yes, for the residents
- yes, for the staff members
- yes, for both residents and staff members
- No

1. When was this PCR testing organized?

*Answer this question only if the following conditions were met:*

*Answer was "yes, for residents" or "yes, both for residents and staff" or "yes, for staff" to question "53 [Self-PCR]" (Did the facility organize its own PCR testing with its own resources even before the testing from the government came in)?*

*Enter a date:*

1. How do you evaluate the availability of the following (personal) protective equipment (for employees) during the Covid-19 epidemic? *

*Choose the appropriate answer for each item:*

|  | definitely insufficient | insufficient | sufficient | definitely sufficient |
| --- | --- | --- | --- | --- |
| disposable gloves |  |  |  |  |
| surgical mouth masks |  |  |  |  |
| P2 filter masks |  |  |  |  |
| disposable aprons (long sleeves) |  |  |  |  |
| face-shields |  |  |  |  |
| safety glasses |  |  |  |  |
| hand alcohol gel |  |  |  |  |

1. Do you feel that staff members were adequately trained to properly use this material? *

*Choose the appropriate answer for each item:*

|  | yes, definitely | rather yes | rather not | no, not at all |
| --- | --- | --- | --- | --- |
| disposable gloves |  |  |  |  |
| surgical mouth masks |  |  |  |  |
| P2 filter masks |  |  |  |  |
| disposable aprons (long sleeves) |  |  |  |  |
| face-shields |  |  |  |  |
| safety glasses |  |  |  |  |
| hand alcohol gel |  |  |  |  |

1. Was additional training organized for employees as a result of one or more of these measures taken?

*Choose one of the following answers*

- yes, for everyone
- yes, but not for everyone
- No

1. Approximately when was the first additional training organized for employees?

*Answer this question only if the following conditions are met:*

*Answer was 'yes, but not for all ' or 'yes, for all ' to question '57 [Training]' (Was additional training organized for employees as a result of one or more of these measures taken?)*

*Enter a date:*

1. During the Covid-19 epidemic, was daily temperature screening implemented among employees? *

*Choose one of the following:*

- Yes
- No

1. From when (approximately) was daily temperature screening of employees introduced?

*Answer this question only if the following conditions are met:*

*Answer was "Yes" to question "59 [Temp Employees]" (During the Covid-19 epidemic, was daily temperature screening implemented among employees?).*

*Enter a date:*

1. What was the policy regarding work and return to work for employees with Covid-19 symptoms?

*Multiple answers possible*

*Choose all of the options that apply to you:*

- Employees stayed home for at least 7 days and resumed only after at least 3 symptom-free and fever-free days
- Workers stayed home for at least 14 days
- Workers were tested before resuming
- Workers were asked to come to work as soon as they were able to do so
- Employees were asked to come to work, but only in a Covid department
- The sick bill from the attending physician was followed
- Other:

1. Since the beginning of the epidemic, have you taken any other specific measures to prevent the infection and spread of coronavirus? *

*Choose one of the following:*

- Yes
- No

1. What were these additional measures?

*Answer this question only if the following conditions are met:*

*Answer was "Yes" to question "62 [MeasuresOther]" (Since the beginning of the epidemic, have you taken any other specific measures to prevent the infection and spread of the coronavirus?).*

*Enter your answer here:*

1. Did you believe there was enough expertise in your facility ( in you or any of your staff) to manage the Covid-19 pandemic?*

*Choose one of the following answers*

- yes, absolutely yes
- yes, previously yes
- no, not before
- no, absolutely not

1. Have you called on expertise from another facility (e.g., expert in care hygiene from another nursing home, hospital hygienist from a hospital, family physician with specific expertise, “Doctors Without Borders”,...)?*

*Choose one of the following options:*

- Yes
- No

1. What is the highest level of qualification you have obtained and in which field was it? *

*Enter your answer here:*

1. Are there any issues or comments that you believe are relevant to us that you would like to share with us?

*Please enter your response here*

We would like to remind you that for the further good conduct of the study, it is necessary to upload the resident and staff list as soon as possible (and certainly before the Liantis nurse comes to sample on site) in tool provided for this purpose, in order to select and number the study participants.

We sincerely thank you for your participation in this study!

Send your survey.

Thank you for your participation in this survey.
