# Supplementary material for: Exploratory study of risk factors related to SARS-CoV-2 prevalence in nursing homes in Flanders (Belgium) during the first wave of the COVID-19 pandemic
Source: PLoS One. 2023 Oct 5;18(10):e0292596. doi: 10.1371/journal.pone.0292596 (PMC10553833; doi:10.1371/journal.pone.0292596)
Supplement: S1 Table — aused in unchanged form bnormalization with the total number of residents cnormalization with the number of questions dcombining two variables into one new variable with new factor levels. (DOCX) [file pone.0292596.s002.docx]

| **Risk factor** | **Question(s)** | **Type of variable** |
| --- | --- | --- |
| **Risk factors related to the community** | | |
| Urbanisation | B.5 ^a^ | Categorical variable - 3 answer options (city, rural, peripheral area) |
| **Risk factors related to NH residents characteristics** | | |
| Ratio females | B.3 & D.1 ^b^ | Continuous variable between 0 and 1, representing the total number of female residents divided by the total number of residents |
| Ratio of older residents (>= 85 years) | B.3 & D.2 ^b^ | Continuous variable between 0 and 1, representing the total number of older (>= 85 years) residents divided by the total number of residents |
| Ratio of residents with dementia | B.3 & D.3 ^b^ | Continuous variable between 0 and 1, representing the total number of residents with dependency class Cd (the residents with dementia) divided by the total number of residents |
| **Risk factors related to NH management and performance** | | |
| Type of organization (organization type) | B.1 ^a^ | Categorical variable - 3 answer options (public, private, non-profit) |
| PCR testing capacity, when needed? (PCR-capacity) | E.25 ^a^ | Categorical variable - 4 answer options  Yes, but only on own resources  Yes, with the government test rounds  Yes, with the government test rounds and on own resources  no |
| Availability of personal protective equipment (surgical & FFP2 masks, safety glasses, faceshields) (PPE: masks and eye/face protection) | E.28 ^c^ | Ordinal variable based on the sum score of the separate questions (referring to the availability of chirurgical and FFP2 mouth masks, face-shields and goggles - scored between 1=definitely insufficient; 2=insufficient: 3=sufficient; 4=definitely sufficient) treated as continuous after division by the number of questions. |
| Availability of personal protective equipment (gloves, aprons and disinfectant alcohol) (PPE: gloves, aprons and disinfectant alcohol) | E.28 ^c^ | Ordinal variable based on the sum score of the separate questions…(referring to the availability of gloves, apron, disinfection alcohol - scored between 1=definitely insufficient; 2=insufficient: 3=sufficient; 4=definitely sufficient) -> as continuous |
| Daily cleaning and disinfection of rooms with a product active against SARS-CoV-2 from the beginning of the pandemic? (room cleaning) | E.16 ^a^ | Dichotomous variable - answer options: yes/no |
| Feeling of being adequately trained to use personal protective equipment (trained to use PPE) | E.29 ^c^ | Ordinal variable based on the sum score of the separate questions… (referring to the feeling of being adequately trained for the use of chirurgical and FFP2 mouth masks, aprons, face-shields, goggles and disinfection alcohol - scored between 1=no, not at all; 2= rather not: 3= rather yes; 4= yes, definitely) -> as continuous |
| **Risk factors related to the building** | | |
| Year of construction of the building | B.6 ^a^ | Continuous variable |
| Combined variable related to the degree of natural ventilation | B.15 & B.16 ^d^ | Ordinal variable with four possible outcomes from 0 to 3  0= No ventilation of rooms & common rooms  1= Ventilation of rooms (by opening windows) & regular ventilation of common rooms  2= Ventilation of rooms (by opening doors) & regular ventilation of common rooms  3= Ventilation of rooms (by opening windows and doors) & continuous ventilation of common rooms |
