# Supplementary figures and images for: Exploratory study of risk factors related to SARS-CoV-2 prevalence in nursing homes in Flanders (Belgium) during the first wave of the COVID-19 pandemic
Source: PLoS One. 2023 Oct 5;18(10):e0292596. doi: 10.1371/journal.pone.0292596 (PMC10553833; doi:10.1371/journal.pone.0292596)

**
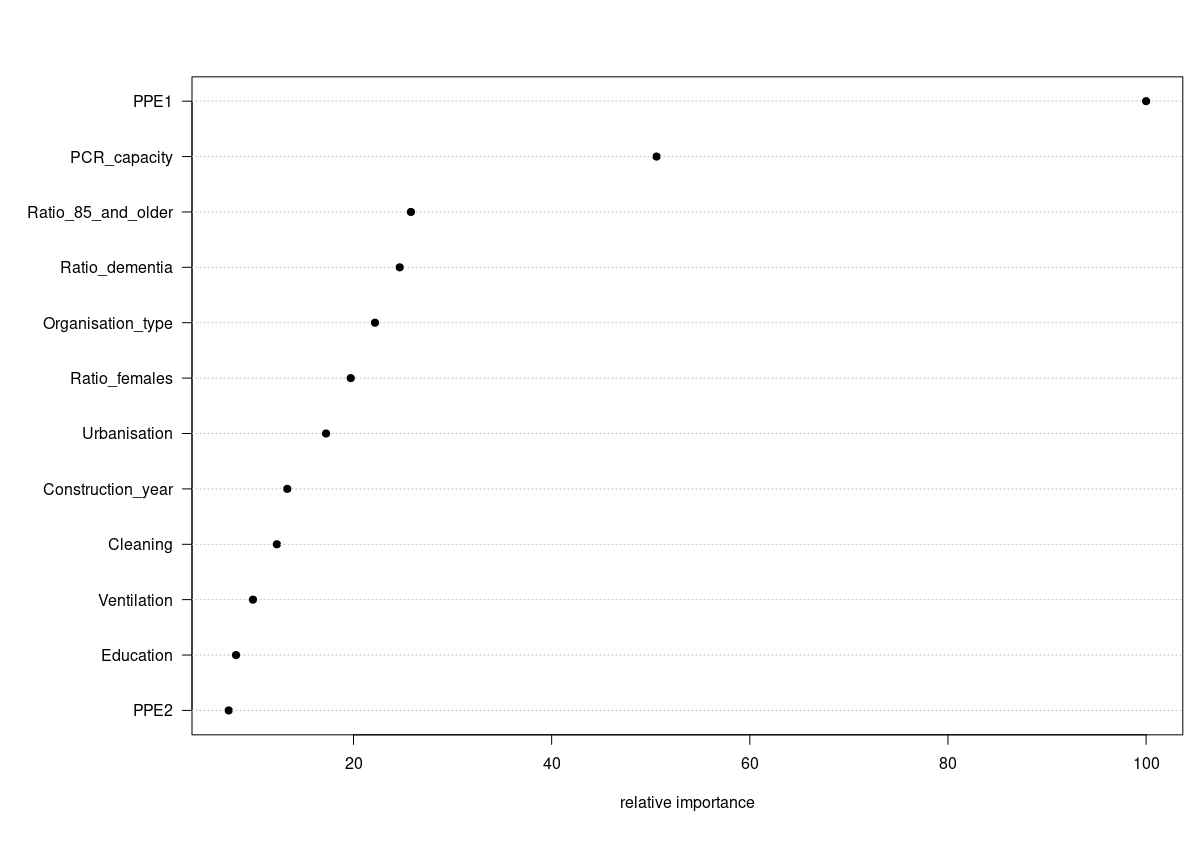
**

Supplement: S1 Fig — (DOCX) [file pone.0292596.s003.docx]
