# Supplementary material for: Exploratory study of risk factors related to SARS-CoV-2 prevalence in nursing homes in Flanders (Belgium) during the first wave of the COVID-19 pandemic
Source: PLoS One. 2023 Oct 5;18(10):e0292596. doi: 10.1371/journal.pone.0292596 (PMC10553833; doi:10.1371/journal.pone.0292596)
Supplement: Attachment — Submitted filename: Response to reviewers .docx [file pone.0292596.s004.docx]

We have read all the comments with great attention and are grateful for the time and effort put in by the reviewer, to help us improve our manuscript. We made changes throughout the paper, considering all recommendations and suggestions. We hope these changes are satisfying and the paper will now be considered to be more in line with expectations and eligibility norms.

Please find below our answers (in blue) to the questions and remarks (in black text) of the reviewers. In the manuscript, the changed sections are marked via track changes.

Reviewer #1: This is a great example of a sound study design and statistical analysis that made use of caution and proper scientific methods to conduct quality research despite unforeseen consequences. The study has been affected by the rapid arrival of Covid-19 wave 2 in the setting, and the anticipated sample size had to be significantly diminished. The authors fully describe this and altered the analysis plan accordingly.

Methods:
1. [lines 105-108] The authors should further describe how the 56 anticipated NH were selected. Was it geographically? How do the 20 that could be included differ from the ones who could not be sampled? What was the acceptance rate? What differences between sampling criteria / available data for those who accepted vs. Refused? This should all be reported and discussed in the manuscript to enable the reader to assess selection bias and internal/external validity of the study results.

We agree that this is important information: we had during the draft of this paper doubts to insert it or to make a referral to the former paper which is about seroprevalence. In order to be concise, it was initially decided to make a reference to the former paper. We now adapted this and inserted this information.

The selection and recruitment was indeed made to obtain a representative geographical sample of the Belgian NH, after that sample size calculations demonstrated that 80 NH across Belgium were needed.

Under the heading ‘study design’, the following sentences were added about the sample size calculation.

*To cover the purpose of both study objectives, sample size calculations demonstrated that 80 NH, with each 60 participants, across Belgium were needed. To anticipate non-response, 100 NH were initially selected.*

Under the heading ‘selection and recruitment of NH’, the following sentences were added:

*The NH were selected from a database of Liantis, a Belgian external occupational health service. A subset of 210 NH employing at least ten staff members was used. In order to obtain a representative geographical sample of the Belgian NH, the 100 NH were chosen according to the true proportion of NH per province in Belgium, based on data available in the Crossroads Bank for Enterprises.*

The gathering of information through the questionnaires started at the same moment as the blood sampling. So, we actually have no questionnaire information (risk factors) from the NH that could not be recruited/sampled. This means that we cannot examine if there are differences between those sampled and the ones that could not be recruited due to the second wave.

Acceptance rate was 37.0%: we contacted 54 nursing homes from the selected ones in the Flemish region, from which 20 were willing to participate. Main reasons for non-participating were:

- are facing Covid cases among residents or staff at the time of study (7 NH)
- want that everyone in the NH could be tested (5 NH)
- do not want to put time and effort in it (6 NH)
- already participate in another Covid-19 study (2 NH)
- ..

We agree that this is an important shortcoming in our study, which may have caused selection bias. We added this now more explicitly in the limitations section. Nevertheless, we think that in this study, the variability in the outcome is more important than representativity: we want to assess relations between risk factors and an outcome.

*Finally, the upcoming second wave may also have caused a selection in the participating NH: one of the main reasons for refusal to participate was that the NH was confronted with a lot of Covid-19 cases at the time of recruitment. Unfortunately, no comparison can be made in seroprevalence or risk factors between NH which accepted and refused to participate, since we have no questionnaire information nor information about the Covid-19 cases and seroprevalence from these NH. Nevertheless, achieving representativeness is less crucial in analytical studies, were associations are examined.*

*Why representativeness should be avoided. Rothman K, Gallacher J, Hatch E. International Journal of Epidemiology 2013;42:1012–1014.*

2. [line 110] What was the number of residents and staff in each of the sampled NH (min, max, mean, SD, median, IQR)? To which proportion of the median do 60 correspond?

As mentioned in the paper, 60 residents and staff were randomly selected. The proportion of selected residents and staff in each NH reflected the proportion of residents and staff in that NH. In one smaller nursing home, the selection was limited to 45 residents and staff due to limited number of staff. In the table below you can find the numbers of residents (‘res’) and staff (‘hcw’) per nursing home.

The first column is the unique code of the nursing home. So, this means that for the staff, the minimum number of participants per NH is 19, the maximum is 32 and the median is 25, while for residents, the minimum number of participants is 24, the maximum is 41 and the median is 35.

hcw res

155047 28 32

211530 22 38

263630 30 30

304177 26 34

329562 21 24

342601 30 30

407523 25 35

496162 32 28

557732 21 39

609780 25 35

663774 32 28

712356 24 36

727120 25 35

801509 24 36

804715 24 36

820275 24 36

842379 28 32

873234 26 34

984348 19 41

990084 22 38

Fivenum HCW: [1] 19 23 25 28 32

Fivenum Residents: [1] 24 31 35 36 41

We added the following sentence below the heading ‘selection of staff and residents’ in the method section:

*This selection procedure resulted in median number of staff participants of 25 (minimum: 19, maximum: 32) and a median number of residents of 35 (minimum: 24, maximum: 41).*

3. [line 114] Why were residents living in assisted facilities excluded? What are the potential impacts of this exclusion to the applicability of the results? How was the prevalence and outcomes of Covid-19 different among those in assisted living facilities vs. not?

This was a subject that was extensively discussed between several of the co-authors during the phase of the study design and questionnaire development.

Indeed, we explicitly only focus on nursing homes.

On the one hand, assisted living facilities are also at risk for having high number of infection and worse outcomes, so they deserve to be studied. We refer to a letter to the editor from Norma Coe, in which is stressed that it is not only needed to develop strategies for infection transmission reduction in nursing homes, but also for other long term care facilities, such as assisted living facilities. [1]

On the other hand, in the reporting of numbers of Covid-19 cases in Europe, no distinction is made and numbers of all long term care facilities are taken together. The European Centre for Disease Prevention and Control reports numbers for ‘long-term care facilities’ which actually include both nursing homes and assisted living facilities. [2] So, it is rather difficult to find references for Covid-19 cases and mortality specifically for this type of long-term care facilities. Nevertheless, several case reports suggest also higher numbers of Covid-19 cases. [3,4]

Additionally, there are some fundamental differences in the risk factors for SARS-CoV-2 seropositivity in comparison with nursing homes. In this context, we can refer to an Editorial in which 7 key areas in which assisted living facilities differ from nursing homes are described with impact on the prevention and implication of infectious diseases. [5] Although the authors describe the American situation, at least some of the topics are recognizable for the Belgian situation. First, the authors describe that the focus in assisted living facilities is more on the social aspect (promoting participation in social activities) rather than on the medical aspects. Individuality and freedom of personal choice are important features. A second difference, according to the authors, is that a large part from the residents is suffering from a cognitive impairment (such as moderate dementia). Third, the authors notice less attention for infection control practices than in nursing homes. Fourth, a large variation in types of assisted living facilities with a high range in beds and care facilities is noticed. A fifth difference is that there is less medical care and nursing presence as in nursing homes. Sixth, the employees in assisted living facilities have limited training. And last, family plays a greater role in the surveillance of the residents. [5]

With respect to the current study, not only the differences with the nursing homes, but also the large variability in infrastructure, organization of care, residents and prevention measures during the Covid 19 pandemic between these assisted living facilities, would have complicated the study design. First we needed enough residents and workers per facility to assess the seroprevalence. When we would have treated an assisted living facility as a separate entity, acquiring this number of workers and residents would have been fairly impossible. A second difficulty is that the survey should question a lot more risk factors due to the variability between assisted living facilities. Moreover, some questions may be difficult to answer on (for instance, some assisted living facilities are not located in the same building; also the wording concerning the use of personal protective equipment is less applicable in the current questionnaire, due to the nature of the contacts such as more basic care and social contacts).

Therefore, we decided, while drafting the questionnaire, explicitly exclude these kind of long-term care facilities.

1. Coe NB, Van Houtven CH. Living Arrangements of Older Adults and COVID-19 Risk: It Is Not Just Nursing Homes. (2020) JAGS. 68, (7): 1398-1399. DOI: 10.1111/jgs.16529)
2. European Centre for Disease Prevention and Control. Surveillance of COVID-19 in long-term care facilities in the EU/EEA, (2021) Stockholm: ECDC.
3. Resnick B, Galik E, Holmes S, McPherson R. The impact of COVID-19 in an assisted living community. (2021) Geriatr Nurs. 42(5): 1151–1155.
4. Yi S, See I, Kent A, et al. Characterization of COVID-19 in Assisted Living Facilities — 39 States, October 2020; pp. 1730–1735. (2020). Morbidity and Mortality Weekly Report. 69(46):1730-1735.
5. Zimmerman S, Sloane P, Katz P, Kunze M, O’Neil K, Resnick B. The need to include assisted living in responding to the COVID-19 pandemic. (2020) JAMDA. 21 572e575. https://doi.org/10.1016/j.jamda.2020.03.024

We added a sentence in the method section under the heading ‘selection of staff and residents’:

*This was done since important differences between NH and assisted living facilities concerning social interactions and caring approach exists, which would have hampered the questionnaire design.*

4. [lines 114-11] What was the refusal rate?

As mentioned in the paper, an additional randomly selected person was invited to participate in case of refusal. This was done by the trusted third party (which was in the majority of cases the NH the director) since the identity of the staff and residents had to be hidden for the researchers: this was asked by the ethical commission. We refer to the methods section, in which this was mentioned: “A confidential counsellor (a family member or a nurse after approval by the family) signed for participants who were incapable to sign the consent form, such as residents with dementia. The authors did not have access to information that could identify individual participants during or after data collection: they only had codes with serology and questionnaire results. Participant lists with names and codes were only available to the director of the NH, acting as a thrusted third party.”

We unfortunately have no information how frequent this had to be done. The only solution to have an idea of the refusal rate is to ask the trusted third parties to give us the lists of participants (if yet available), but then we break the anonymity of the participants. Therefore, we prefer not to do this.

Nevertheless, in the instruction to the trusted third party, we explicitly mentioned that the firstnext person on the list should be asked to participate (and that it was important for the study that they not themselves choose a participant). The sequence in which the persons were ordered on the list was randomly generated by the tool.

This was now added in the limitation section as following:

*A last limitation is that we have no information about the number of residents and staff that refused to participate, nor the reasons for refusal, since this information was gathered by the trusted third party.*

Results:
5. [lines 188-190] The first sentence needs to be clarified. Do the authors mean that 92.2% of the total 1093 individuals included has a valid serological test result? Please rewrite the sentence for clarity. What were the reasons for the absence of a valid serological test in those 7.8%?

We agree that this sentence was not clearly written. Actually, in total 1185 participants were included in the study. From 478 staff members and 615 residents a valid serological test result was obtained which corresponds to 92.2% of the total study population.

We can not exactly know what the exact reason is for the absence of a valid serological test result in 7.8% of the included participants. As mentioned in the answer on the former question, the name list with codes was only available to the trusted third party. On this list, the nurse indicated whether the blood sample was obtained or not (and eventually the reason for not obtaining a valid sample). The laboratory received samples with codes. And the researchers received the questionnaire results with the codes and the blood results with the codes.

The nurses who took the blood samples were instructed to check if the quality of the blood sample was accurate. If not (unable to gather enough blood on the blood card), these samples were not transported to the laboratory.

Below, you can see a couple of slides from the instruction presentation, which was given to these nurses.

Another reason for not obtaining a blood sample may be that the participant agreed to participate, but was absent at the moment of the collection

In line with the remarks of the reviewer, we now changed the section ‘Seroprevalence’:

*From 478 staff members and 615 residents from the 20 NH (1093 of the 1185 included participants which corresponds to 92.2% of the total study population), a valid serological test result was obtained. Main reasons for this missing information were the absence of the participant at the moment of sample collection or the impossibility to obtain enough blood.*

6. [lines 210-225] The authors should consider moving this text to the Methods section.

This section has been replaced to the Methods section ‘statistical analysis’.
